# Fc Gamma Receptors in the Hepatic Sinusoid

**DOI:** 10.1186/1476-5926-2-S1-S23

**Published:** 2004-01-14

**Authors:** Henrik Braathen, Seyed A Mousavi, Trond Berg, Rune Kjeken

**Affiliations:** 1 Department of Biology, University of Oslo, P.O. Box 1050 Blindern, N-0316 Oslo, Norway

## Introduction

Receptors that recognize the Fc-portion of antibodies mediate endocytosis of antibody-containing complexes (ICs) [1,2]. The liver is the main organ for uptake of IC from circulation. Phagocytosis of antibody-coated particles takes place in Kupffer cells (KC) whereas endocytosis of soluble IC takes place both in KC and in liver endothelial cells (LEC) [3,4]. ICs have, in comparison with ligands of the mannose-receptor or the scavenger receptors, been shown to be delayed in the endocytic pathway en route to lysosomes [4,5] suggesting that the ICs and the Fc gamma Rs may use a unique pathway to lysosomes. Whereas the receptors of mannose and scavenger receptors are recycled constitutively between endosomes and plasma membrane, with or without bound ligand [6], the traffic of the Fc gamma R may be controlled by the ligand. Polyvalent ligands (ICs) which bring about receptor cross-linking may direct the receptor-ligand-complex from a recycling route to late endosomes and eventually to lysosomes. The latter pathway will lead to receptor-downregulation [7]. Few studies have been done to determine whether hepatic Fc gamma Rs are subject to downregulation by their specific ligands. If these receptors were lost from KC and/or LEC after uptake of immune complexes a fatal result would be that the liver were left defenseless until new receptors were synthesized. The purpose of the present investigation was to determine whether uptake of polyvalent ligands would bring about a reduction in the number of Fc gamma Rs in rat KC and LEC.

## Methods

### Animals and reagents

Male Wistar rats, weighing 200–300 g, were obtained from M–llegaard and Bomholt (Ry, Denmark). Eagle minimal essential medium, glutamine and penicillin/streptomycin were obtained from Bio Whittaker, INC (Walkersville, MD). Na^125^I was purchased from Isopharma (Kjeller, Norway).

### Preparation and culture of cells

Isolated liver cells and pure cultures of KC and LEC were prepared as described earlier [8]. For all experiments cells were grown on Petri dishes (6 cm in diameter). The mouse peritoneal macrophage cell line J774-A1 was cultured in monolayers in 6-cm dishes with approximately 1.5 – 10^7 ^cells in each dish. DMEM containing 10% fetal calf serum, 1% pen/strep (penicillin 10,000 U/ml, streptomycin 10,000 ug/ml) and 1% L-glutamine was used.

### Radiolabeling and preparation of aggregated IgG

Human IgG was diluted with PBS and iodinated with 40 MBq Na ^125^I in a iodogen coated glass tube in a final volume of 100 microliters. The radioiodination was carried out for 30 min at room temperature and arrested by passage over a Sephadex G-25 PD-10 column. Aggregated human IgG was (AgIgG) prepared by incubating the labeled IgG (1.3 mg/ml) for 45 min at 63 degrees C.

### Antibodies against Fc gamma Rs-receptors

Monoclonal antibodies, mouse anti-rat CD32 and rat anti mouse CD32/16 were from Research Diagnostics Inc, NJ, USA).

## Results

### Uptake and degradation of ^125^IAgIgG in KC, LEC and J774 cells

**Figure 1:**
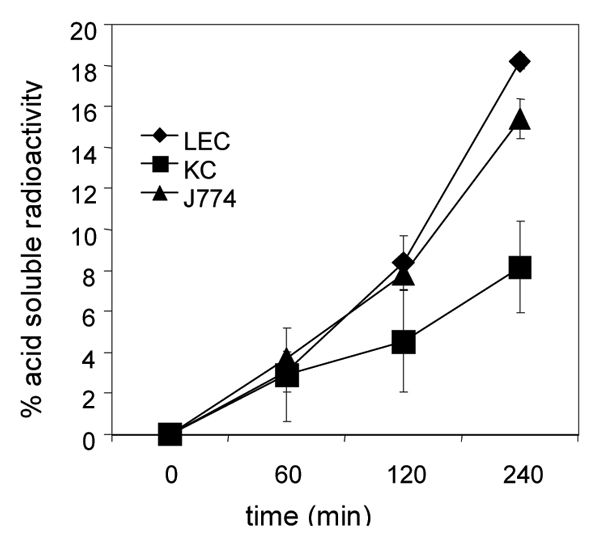
Degradation of ^125^I-labeled AgIgG in LEC, KC and J774 cells. ^125^I-AgIgG (10 micrograms/ml) was added to the cells (10^6 ^cells per ml) incubated at 37 degrees C. Aliquots of medium, removed at the indicated time points, were treated with ice-cold trichloroacetic acid and centrifuged at 1000 – g for 10 min. Acid-soluble radioactivity in the supernatant is presented as % of total acid precipitable radioactivity added to the cells at the start of the incubation. At the end of the incubation (240 min) about 1.5% of the total radioactivity was present in the cells. The results are given as means – S.D. of 3 experiments.

All three cell types took up and degraded ^125^I-AgIgG (Fig. 1). After four hours LEC and J774 cells had degraded about 20% whereas KC had degraded about 10% of the ^125^I-AgIgG added to the cells. Degradation of ^125^I-AgIgG was inhibited by leupeptin indicating that that the process took place in lysosomes (results not shown).

### Antibodies against Fc gamma Rs inhibit binding of ^125^I-Ag IgG to LEC, KC and J774 cells

**Figure 2:**
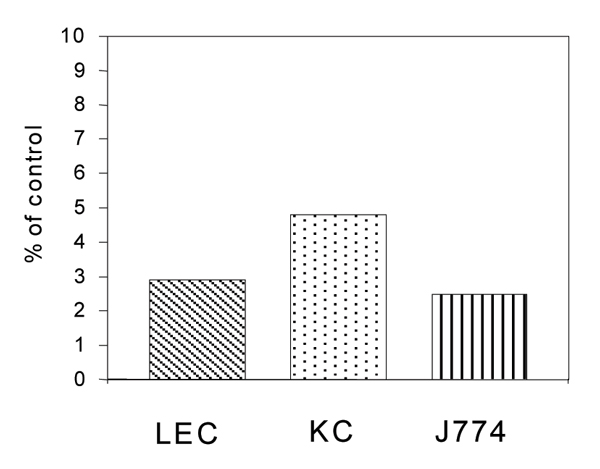
Anti-Fc gamma R IgG inhibits binding of ^125^I-AgIgG. LEC, KC and J774 cells were incubated with mouse anti rat CD32 (KC and LEC) or rat anti-mouse CD16/32 (J774 cells) antibodies (10 micrograms/ml) on ice. ^125^I-AgIgG (10 micrograms/ml) was then added, and cell-associated radioactivity was measured after incubation on ice for 2 hours. The results represent % of control (binding in absence of antibodies) for one typical experiment.

Binding of antibodies to the cells at saturating concentrations almost completely prevented binding of ^125^I-AgIgG (Fig. 2).

### Down-regulation of Fc gamma Rs in LEC, KC and J774 cells

**Figure 3:**
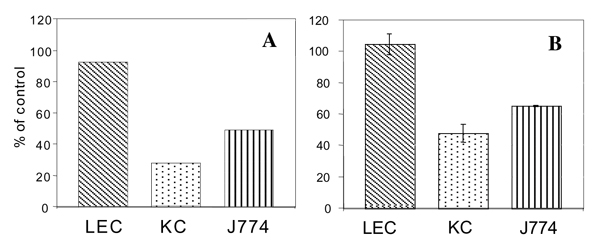
Surface Fc gamma Rs are downregulated in KC and J774 cells but not in LEC. The cells were incubated for 4 hours at 37 degrees C with saturating concentrations of AgIgG (150 micrograms/ml) and then washed and incubated in medium without AgIgG for 2 hours. The cells were subsequently incubated on ice and ^125^I-labeled AgIgG (10 micrograms/ml) (A) or anti-Fc gamma R IgG (5 micrograms/ml) (B) were added. Cell-associated radioactivities were determined after 1 hour.

The cells were incubated with saturating concentrations of unlabeled AgIgG for increasing time intervals. The cells were then separated from the medium and incubated for 2 hours in absence of ligand to internalize surface-bound AgIgG, and subsequently incubated with either ^125^I-AgIgG or ^125^I-anti Fc gamma R antibodies (3B) at 4 degrees C to measure the amount of surface receptors remaining in the cells. Principally similar results were obtained in cells incubated between 2 and 20 hours with an excess of unlabeled AgIgG. Fig. 3 shows the results obtained in cells incubated for 4 hours with unlabeled AgIgG and then for 2 hours in absence of AgIgG. The results obtained using labeled AgIgG or anti Fc gamma R antibodies to determine binding suggested that the number of surface receptors was reduced by about 50% in KC and J774 cells and was unaffected in the LEC.

## Discussion

Fc gamma Rs, following cross-linking by multivalent ligands, may be redirected from a recycling to a lysosomal route [7]. The preliminary data presented in this report suggest that uptake of AgIgG leads to partial downregulation of the Fc gamma Rs in KC and J774 cells but apparently not in LEC. The latter result was unexpected as it suggests that the Fc gamma Rs in these cells are able to release the polyvalent ligand intracellularly and recycle to the plasma membrane. Ligands internalized via the Fc gamma Rs in LEC are degraded efficiently in the lysosomes of these cells [3,4], and it may be calculated that the number of ligand molecules internalized during 4 hours in the LEC is higher than the number of surface receptors. It should be noted though, that an unknown pool of Fc gamma Rs may be present inside the cells from which receptors may be recruited and replace those internalized during the experiment. The mechanisms whereby endocytic receptors are routed to lysosomes or redirected from a recycling to a lysosomal route have been enigmatic. Recent studies have revealed that conjugation of proteins with ubiquitin is a signal for lysosomal sorting [9], and it has been shown that Fc gamma RIIA on leucocytes can be ubiquitylated and that endocytosis (but not phagocytosis) of aggregated IgG seems to require ubiquitylation of the receptor [10]. The different effects of receptor crosslinking in KC and LEC could conceivably be due to differences in receptor ubiquitylation and/or the expression of ubiquitin-binding proteins in the two types of cells in the liver sinusoid.
